# Mechanical Performance of Biodegradable Thermoplastic Polymer-Based Biocomposite Boards from Hemp Shivs and Corn Starch for the Building Industry

**DOI:** 10.3390/ma12060845

**Published:** 2019-03-13

**Authors:** Arūnas Kremensas, Agnė Kairytė, Saulius Vaitkus, Sigitas Vėjelis, Giedrius Balčiūnas

**Affiliations:** Laboratory of Thermal Insulating Materials and Acoustics, Institute of Building Materials, Faculty of Civil Engineering, Vilnius Gediminas Technical University, Linkmenu st. 28, LT-08217 Vilnius, Lithuania; arunas.kremensas@vgtu.lt (A.K.); saulius.vaitkus@vgtu.lt (S.Vaitkus); sigitas.vejelis@vgtu.lt (S.Vėjelis); giedrius.balciunas@vgtu.lt (G.B.)

**Keywords:** polymer binder, corn starch, hemp shivs, biocomposite boards, mechanical performance

## Abstract

Bio-sourced materials combined with a polymer matrix offer an interesting alternative to traditional building materials. To contribute to their wider acceptance and application, an investigation into the use of wood-polymer composite boards is presented. In this study, biocomposite boards (BcB) for the building industry are reported. BcB are fabricated using a dry incorporation method of corn starch (CS) and hemp shiv (HS) treatment with water at 100 °C. The amount of CS and the size of the HS fraction are evaluated by means of compressive bending and tensile strength, as well as microstructure. The results show that the rational amount of CS independently of HS fraction is 10 wt.%. The obtained BcB have compressive stress at 10% of deformation in the range of 2.4–3.0 MPa, bending of 4.4–6.3 MPa, and tensile strength of 0.23–0.45 MPa. Additionally, the microstructural analysis shows that 10 wt.% of CS forms a sufficient amount of contact zones that strengthen the final product.

## 1. Introduction

Wood and polymer-based composites are low-carbon and environmentally-friendly materials. They have many advantages, such as a lightweight, corrosion resistance, dimensional stability, and recyclability, and are widely used in outdoor construction, logistics, decoration, and so on [1].

Even though hemp concrete is characterised by sufficient thermal insulating properties (from 0.05 to 0.13 W/(m·K) [2]), it is not resistant to mechanical impact. After conducting compressive strength tests on hemp shiv (HS) and lime-based composites, [3,4] obtained values of 0.20–0.50 MPa and from 0.40 to 1.2 MPa, respectively. Other authors [5] tested composites from HS and different classes of hydraulic lime. The experimental results of the composites, which were hardened for 28 days at 20 °C and 50% relative air humidity, showed that the compressive strength of such composites ranges from 0.10 to 0.31 MPa when the density is 460–480 kg/m^3^. Authors [6] tested composites from HS and slaked, as well as hydraulic lime. The results showed that the compressive strength varied from 0.15 to 0.20 MPa. Taking into consideration bending strength, according to literature [7,8], it varied from 0.20 to 0.32 MPa. However, no research on tensile strength of hemp and lime-based composites was conducted. Due to low mechanical durability, materials of this type cannot be used for bearing structures or their element production; therefore, they must be combined with wooden or other types of bearing frame, or used as regular thermal insulating materials [9].

Scientists [10] developed and tested composites from HS and used as a binder a mixture of hydraulic lime/Portland cement CEM Ι 42.5 R, as well as sole Mg–O cement. When a binder from the lime/Portland cement mixture was used, the density of the obtained composites ranged from 808 to 1156 kg/m^3^, and the compressive strength from 0.70 to 0.80 MPa, while Mg–O cement exhibited values of 1113–1285 kg/m^3^ and 1.6–2.2 MPa. Additionally, authors [11] conducted tests on composites from complex mineraliser-treated HS and cement-based binder. The results for the compressive strength surprisingly increased from 1.8 to 8.0 MPa, while the density varied from 819 to 1079 kg/m^3^.

Although the previously mentioned binders and their mixtures possess sufficient mechanical performance, their production requires high energy demands and emits high amounts of CO_2_, and they are not renewable. However, such binders can be replaced by ecological and renewable alternatives. An example of such a binder is sapropel—an excavated material obtained from the bottom of lakes and swamps that mainly consists of plankton, benthos, algae and other hydrophyte remains layered together with sand, clay and limestone particles [12,13,14]. Scientists [15] developed and tested composites from different compositions made of HS aggregate, sapropel binder and fine cellulose fibres. The final products had a density of 210–410 kg/m^3^ and compressive stress at 10% of deformation of 0.61–2.1 MPa. Further research with a sapropel binder was conducted by [16] who obtained composites with compressive strength ranging from 0.01 to 0.15 MPa, bending strength from 0.007 to 0.031 MPa and density from 112 to 196 kg/m^3^.

Another type of ecological binder and aggregate is starch/water paste and natural fibres from renewable resources [17,18,19]. Regretfully, low resistance to water impact and alteration in mechanical properties at varying environmental conditions, limit wider starch application in bioplastic production [17,20]. Compared to composites for which production lime, cement or sapropel-based binder were used, the mechanical properties of such composites are worse, with compressive stress at 25% of deformation varying from 0.57 to 0.63 MPa and tensile strength from 0.080 to 0.11 MPa [21]. Such low values of mechanical properties were obtained due to application of CS/water paste on dry (non-surface-treated) HS which abruptly absorbed water from CS/water paste; therefore, non-homogeneous distribution of binder was observed. For the improvement of the interaction behaviour between HS aggregate and CS/water paste binder, researchers [22] decided to test composites from NaOH-treated and non-treated HS. The non-treated HS-based composite had compressive strength equal to 0.40 MPa, while the treated HS-based one had twice the strength. Overall, NaOH treatment led to bending strength, which had a value of 0.25 MPa. The effect of NaOH can be explained by its ability to activate HS surface by degrading extractives. Therefore, in order to obtain mechanically stronger products, surface treatment is advisable.

Most of the above-discussed processing of composites includes additional HS treatment with non-ecological NaOH. Even though lime, cement and gypsum-based products are in excellent agreement with the basic requirements for building materials, the current demand is for more ecological and energetically efficient materials. As a literature review shows [17,18,19,20,21,22], CS is an excellent alternative to traditional binding materials. However, CS/water paste-based products need to be improved in order to meet more stringent requirements for mechanically stronger building materials. Therefore, the aim of this study is to use new, environmentally friendly methods of HS treatment with 100 °C water to replace chemical modification of aggregates’ surface (NaOH, titanates, silanes) which is used to improve interfacial adhesion between HS and CS binder. Additionally, dry application of thermoplastic CS powder into a forming mixture for the preparation of BcB was applied to assure even distribution. Such products are fully ecological and have improved mechanical performance, which meets the basic requirements of fibreboards standard EN 622-4 [23].

## 2. Materials and Methods

### 2.1. Raw Materials

For the preparation of biocomposite boards (BcB), fibre HS aggregate (obtained from local farmers (USO 31 species), Rokiskis region, Lithuania) arising from a hemp fibre separation process was used. In order to conduct the tests, the following HS fractions (the particle size range) were chosen: 2.5/5 (particles size from 2.5 to 5 mm), 5/10 (particles size from 5 to 10 mm), 10/20 (particles size from 10 to 20 mm) and 2.5/20 mm (particles size from 2.5 to 20 mm). Additionally, the shredded fraction obtained from milling a 2.5/20 mm fraction (up to 5.6 mm) was used. As a binder, corn starch (CS) (“Roquette”, Lestrem, France) with a bulk density of 550 kg/m^3^, compressibility—40%, amylose content—26%, moisture content—11.4% and gelatinization temperature—62 °C was chosen and used at 10, 20, 30, 40 and 50 wt.%. The viscosity is not relevant due to a dry incorporation of CS into wetted HS. The tensile strength of hardened sole starch/water paste—1.40 ± 0.21 MPa [24].

### 2.2. Forming Process

BcB were formed from different HS fractions, CS and water. Shredding of the 2.5/5 mm fraction was conducted with a laboratory shredder (self-made, Vilnius, Lithuania) having a power of 1.1 kW and blade rotational speed of 2800 rpm. The aim of shredding was to fibre HS particles to fine fibres. While fibreing, it was noticed that the process was not successful due to the high amount of dust and the shape of the particles changing only slightly. Therefore, HS were poured with 100 °C water and left for 2 h. Further, wetted HS were then were shredded for 60 s.

All fractions were treated with 100 °C water and left for 2 h, after which they were drained for 10 min in order to eliminate an excess of water. Into water treated HS, powder type CS (without any pre-treatment) was dosed through a 0.63 mm-sized sieve. All HS fractions were mixed with different amounts of CS each. Totally, 30 compositions were formed (Table 1). The obtained mixtures were then thoroughly mixed for no less than 3 min until a homogeneous mass was obtained.

**Figure 1:**
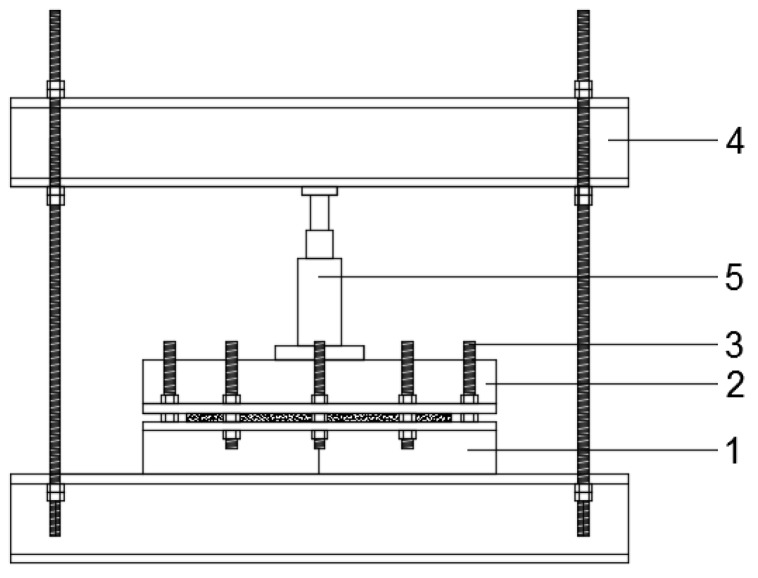
Forming setup of biocomposite boards (BcB): 1—bottom part of mould; 2—upper part of mould; 3—screws; 4—stand; 5—hydraulic jack.

BcB were formed using a metal mould, as presented by a graphical sketch in Figure 1. On the bottom part of the metal mould, which is lubricated with oil, a wooden frame is added. Furthermore, the forming mixture is distributed through the whole frame. Then, the mixture is trampled down with wooden scantling in order to obtain the initial form. After that, the upper part of the mould is added onto the bottom part and both of them are screwed together. The whole setup is then put on the stand, and a hydraulic jack is set on the upper part of the mould. The mixture is compressed up to 40 vol.%.

After the loading, BcB are further thermally hardened in a ventilated oven. Thermal hardening consists of three stages: temperature increase (160 °C in 1 h), temperature maintenance (160 °C for 6 h) and temperature reduction (heating is turned off). After the thermal hardening, the mould with a hardened product is left in the thermal processing oven until it cools down to environmental temperature. Then, hardened BcB are demoulded and cut into specimens.

### 2.3. Tests Methods

Compressive stress at 10% of deformation was tested according to the EN 826 method [25] using a computerised machine H10KS (Hounsfield, Surrey, UK) with a maximum loading force of 10 kN, a loading accuracy of ± 0.5% and a loading speed accuracy of ± 0.05%. Three specimens for each composition with a size of 50 × 50 × *d* mm^3^ (*d*–thickness of specimen) were prepared. Before the test, specimens were conditioned for not less than 6 h at 23 ± 5 °C. Then, the specimen is aligned onto the bottom support and loaded with an initial loading of 250 ± 10 Pa. The loading speed during the tests is 0.1*d* min^−1^ and the specimen is compressed until 10% of deformation.

The bending strength of the BcB was determined in accordance with the EN 310 method [26]. For the test, equipment consisting of two parallel cylinder supports, which have a length greater than the side of a specimen, and diameters of 15 ± 0.5 and 30 ± 0.5 mm, were used. The test was conducted using the same computerised machine as for compressive stress determination for three specimens with a size of (20*d* + 50) × 50 × *d* mm^3^. Before the test, specimens were conditioned at 20 ± 2 °C and 65% ± 5% relative air humidity until constant mass was achieved.

The tensile strength perpendicular to the specimen surface was tested based on the EN 319 method [27]. The test was conducted using the computerised machine used for compressive stress and bending strength determination. Three specimens for each composition with a size of 50 × 50 × *d* mm were prepared. Before the test, specimens were conditioned at 20 ± 2 °C and 65% ± 5% relative air humidity conditions until constant mass was achieved.

The density was determined according to EN 1602 [28] for specimens which size was the same as for mechanical properties testing. Three density ranges were obtained, i.e., separately for compressive stress, bending and tensile strength.

The structure of the BcB was studied using scanning electron microscopy (SEM) with a JEOL SM–7600F (JEOL Ltd., Tokyo, Japan). Before the SEM analysis, the BcB were sputter coated with a thin gold layer under vacuum using a QUORUM Q150R ES (Quorum Technologies Ltd., Lewes, UK).

Experimental analysis of the obtained test data was conducted using mathematical and statistical methods, during which standard deviations were evaluated, and distribution functions and parameters were determined using the software STATISTICA (8.0). For the determination of the optimal relationship between *X* and *Y*, linear and non-linear correlation methods were used. According to the obtained determination coefficient, it is possible to conclude a relationship between the two parameters. When the value of the correlation square ratio is >0.9, the relationship is very strong, when it is in the range of >0.7–0.9 it is strong, when it varies from 0.5 to 0.7 it is averagely strong and when it is <0.5 it is weak [29]. In order to evaluate the scattering of experimental data on both sides of the regression line, the average square deviation *S*_r_ was determined.

## 3. Results and Discussion

### 3.1. Compressive Strength of BcB

**Figure 2:**
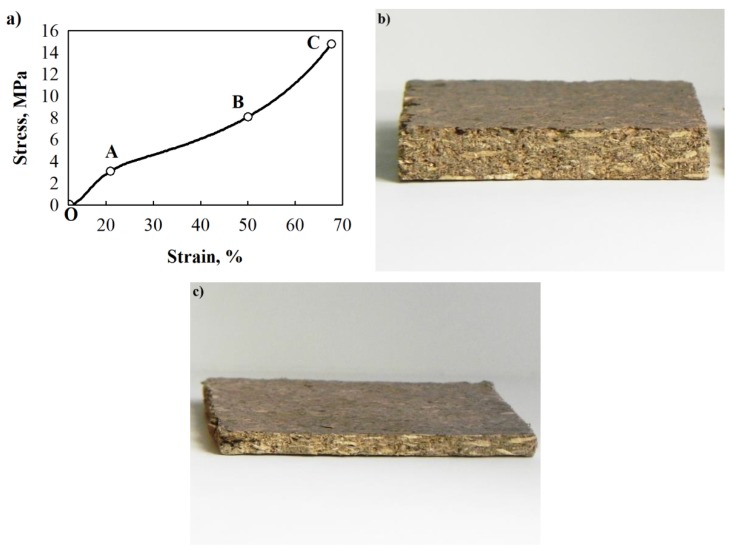
Peculiarities of short-term compression of BcB: (**a**) compression curve “stress–deformation”; (**b**) non-compressed BcB 50 × 50 × 10 mm^3^; (**c**) up to 70% relative deformation compressed BcB 50 × 50 × 3 mm^3^.

Most thermal insulating materials under compression do not show an evident fracture limit—specimens do not fracture but densify. The same manner has been observed for some porous thermal insulating materials and lime and HS-based composites [30]. When such materials are compressed, the conditional strength limit is determined. Dots O, A, B and C in BcB from the HS aggregate and CS binder compression graph (Figure 2a) designate a smooth transition into other mechanical states. The OA section shows linear or close to linear relation between deformation and loading. Furthermore, compared to the OA section, the AB section shows an increase in deformation when the loading is marginally increased. The third zone (BC section) presents a reduction in deformation while the stress increases—densification of the material is observed.

Figure 2b shows the 50 × 50 × 10 mm^3^-sized non-compressed specimen of BcB, while Figure 2c depicts the up to 70% compressed specimen which has the final size of 50 × 50 × 3 mm^3^. It can be seen that the specimen densifies but does not fracture. 

This way, when the compressive stress is determined for BcB, the limiting compressive strength is not obtained; therefore, it has to be taken into account that permissible compressive loadings are specified. Hereby, normative references for thermal insulating materials designate compressive strength as compressive stress at 10% of deformation (based on thickness).

**Figure 3:**
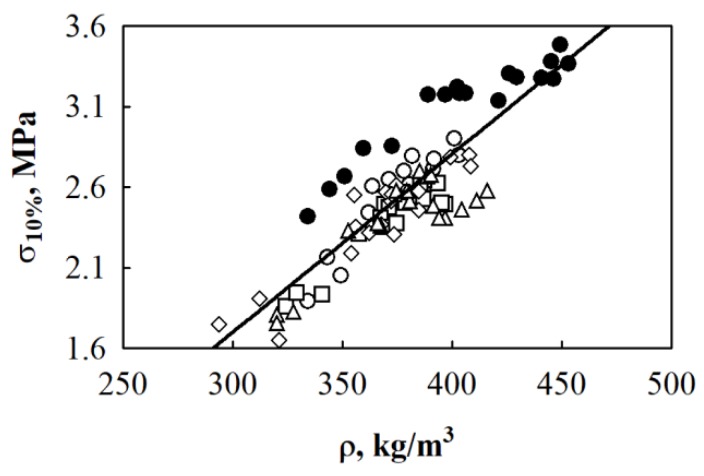
Relationship between compressive stress at 10% deformation and density where designated dots present experimental data for BcB from HS fraction, mm: ○—5/10; □—10/20; ◊—2.5/20; ∆—2.5/5; ●—shredded shivs; (───)—regression line.

The apparent density is an important parameter for describing the mechanical properties of building materials. Therefore, it is important to evaluate its impact on BcB from HS and CS. The dependence of BcB compressive stress at 10% deformation on density is presented in Figure 3. Based on the experimental data, the relation between compressive stress and density is determined and can be approximated by the regression equation (Equation (1)) with standard deviation *S*_r_ = 0.196 MPa (*n* = 90) and correlation square ratio η^2^*_yx_* = 0.756:

(1) $$\sigma_{10\%}^{}=0.011077\rho -1.623$$

where σ_10%_ is the compressive stress at 10% deformation, MPa and ρ is the density of BcB, kg/m^3^.

According to the chosen mathematical model, the obtained correlation square ratio is η^2^*_yx_* = 0.756 and it shows that 75.6% of changes in compressive stress are determined by the change in BcB density.

It was calculated that the average density obtained for BcB with HS aggregate and different amounts of CS binder varies from 319 to 408 kg/m^3^. However, authors [21] obtained HS and starch-based biocomposites within the similar binder amount interval with less than half the density. Additionally, scientists [31] present literature values for hemp-based composites with a varying density from 351 to 627 kg/m^3^, which proves that the parameter is dependent on the formation technology, i.e., loading conditions and HS treatment.

**Figure 4:**
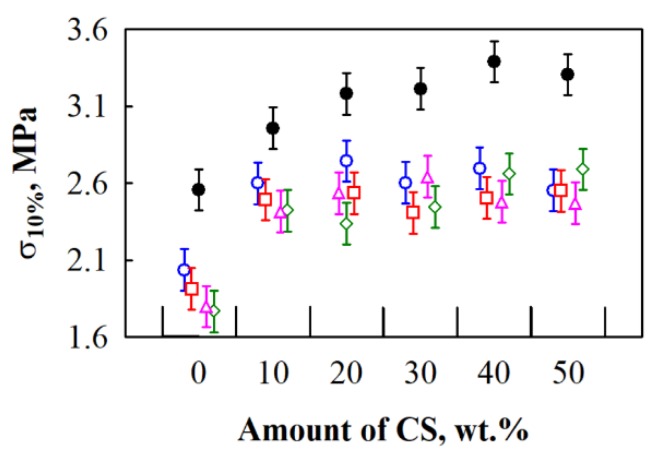
Impact of various amounts of corn starch (CS) binder and different hemp shiv (HS) fractions on compressive stress of BcB: ○—5/10 mm; □—10/20 mm; ◊—2.5/20 mm; ∆—2.5/5 mm; ●—shredded HS.

After conduction of the experimental data analysis (Figure 4), it is determined that differences occurring due to the interaction between varying amount of CS and different HS fractions, are relatively small. The highest compressive stress values are obtained for BcB from shredded HS and CS binder. When the amount of CS varies from 10 to 50 wt.%, the obtained compressive stress ranges insignificantly, it is within the error range (from 3.0 to 3.3 MPa). Moreover, compared to the control BcB without a binder, the greatest increment, i.e., ~15.6%, is observed for BcB from shredded HS and 10 wt.% of CS.

Contrary to the obtained results for BcB with shredded HS, BcB from 5/10, 10/20, 2.5/20 and 2.5/5 mm HS fractions are characterised by the lowest compressive stress values. The parameter, when from 10 to 50 wt.% of CS is used, averagely varies from ~2.5 to ~2.6 MPa. The obtained values are similar to the ones presented by [32] who additionally treated HS with NaOH, Ca(OH)_2_ and ethylenediamintetracetic acid. It means that the same or even better values may be obtained without further treatment of the aggregate. Results regarding the control BcB from all fractions, except the shredded one, and without a CS binder, are lower, the average value of compressive stress is ~1.9 MPa. This can be attributed to the assumption made by [5] that when the proportion of smaller HS particles (in this case shredded ones) is larger, they are better coated by the binder during the production process.

**Figure 5:**
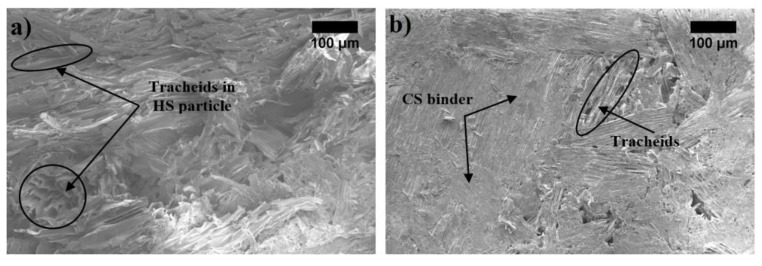
Microstructure of BcB (magnification ×100): (**a**) without CS binder; (**b**) with CS binder.

As can be seen, Figure 5a presents the microstructure images of BcB without a CS binder and Figure 5b for BcB with a CS binder. Figure 5a shows that HS particle treated at 100 °C has rough and dishevelled surface. Hot water treatment leads to breaking microfibril bundles and defibrillation. The recent study of [33] proved the defibrillation effect on piassava fibres. Therefore, better interfacial adhesion is ensured between aggregate and thermoplastic binder. The addition of CS binder into forming mixture allows overlaying of tracheids on the surface of HS, then, during the thermal treatment (BcB hardening process), it forms links that strengthen the contact zones between HS.

In the SEM images (Figure 5b) of the BcB with a binder, it is noted that the HS are fully enclosed by the CS matrix and a considerable adhesion occurs in the interface region between the two components. This result is in accordance with the performance of previously discussed compressive stress tests results and data obtained by [34], which show a good interaction between aggregate and a binder.

### 3.2. Bending Strength of BcB

**Figure 6:**
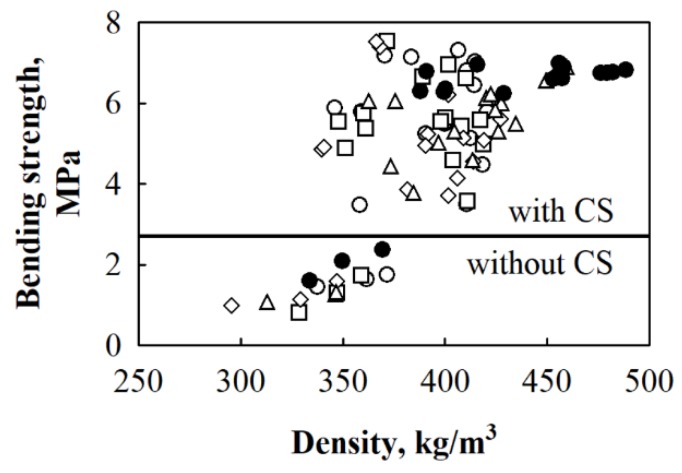
Relationship between bending strength and density where designated dots present experimental data for BcB from HS fraction, mm: ○—5/10; □—10/20; ◊—2.5/20; Δ—2.5/5; ●—shredded HS; (───)—separation line for results obtained from specimens with and without CS binder.

**Figure 7:**
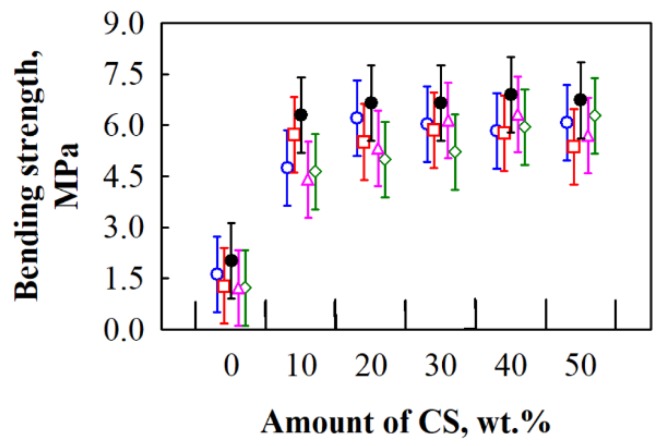
Impact of various amounts of CS binder and different HS fractions on bending strength of BcB: ○—5/10 mm; □—10/20 mm; ◊—2.5/20 mm; ∆—2.5/5 mm; ●—shredded HS.

In order to use BcB from fibre HS aggregate as thermal insulating-structural materials, bending strength, which is an extremely important parameter, should be determined. Based on the results obtained, it may be possible to decide the product’s durability during transportation, installation, exploitation under specific conditions, as well as application in ceilings, external layers of three-layered boards, envelopes and so on. Therefore, the results of bending strength for BcB from different fractions of HS and varying amounts of CS are presented in Figure 6 and Figure 7. Basically, Figure 6 presents the dependence of bending strength on BcB density.

After conducting an analysis of the experimental data (Figure 7), it can be seen that differences occurring due to the interaction between different amounts of CS and various HS fractions are relatively small.

The average value of bending strength for BcB from HS aggregate and CS binder is ~1.5 MPa. It is noticed that different amounts of CS and various HS fractions do not impact the final value of bending strength, i.e., for all BcB, it changes insignificantly. When CS binder is added from 10 to 50 wt.%, the value of bending strength increases from ~5.2 to ~6.0 MPa. According to experimental data, it can be stated that the highest increment in parameter, i.e., 3.5 times, is observed for BcB from various HS fractions and 10 wt.% of CS binder. The more obvious increase in bending strength of BcB is observed in [35] with an increasing amount of CS. However, the authors obtained values varying from 0.03 to 0.13 MPa, while the current study investigates much stronger BcB. Such a significant difference may be due to the different incorporation of starch and loading conditions, i.e., the authors used starch solution, while the current study investigated a dry method. In contrast, the information regarding the loading conditions during production was not presented.

**Figure 8:**
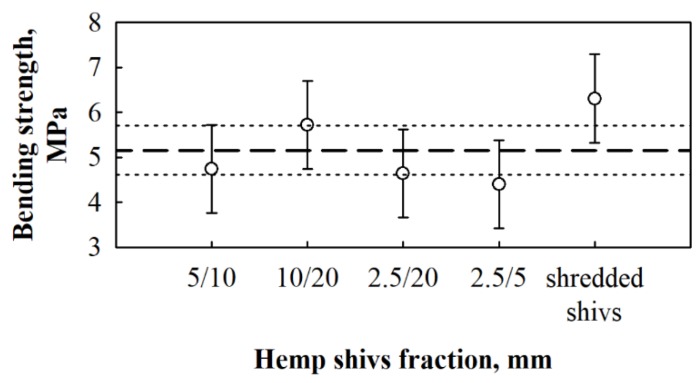
Impact of different HS fractions and 10 wt.% of CS binder on bending strength of BcB. (- - - - -)—the average values of the whole bending strength results; (·········)—confidence interval with 95% reliability.

Whereas the bending strength values of BcB from various HS fractions and 10 wt.% of CS binder are the highest, Figure 8 analysis if the average values fall into the confidence limits where the average value of bending strength for the whole sample is ~5.2 MPa and the standard deviation, *S*_r_ = 0.99 MPa (*n* = 15).

It can be stated that the average bending strength value falls into the 4.6 ≤ σ_b_ ≤ 5.7 MPa interval. Even though all values fall into the interval, BcB from shredded HS can be distinguished due to the average value of bending strength, which is higher than the upper confidence limit. Therefore, the assumption can be made that the bending strength for shredded HS-based BcB is relatively higher than for ones from non-shredded HS. According to the density, BcB from shredded HS fraction can be referred to as low density boards. Although, the density is low, the bending strength, as per given in the study of [36], is almost the same as for medium density boards.

**Figure 9:**
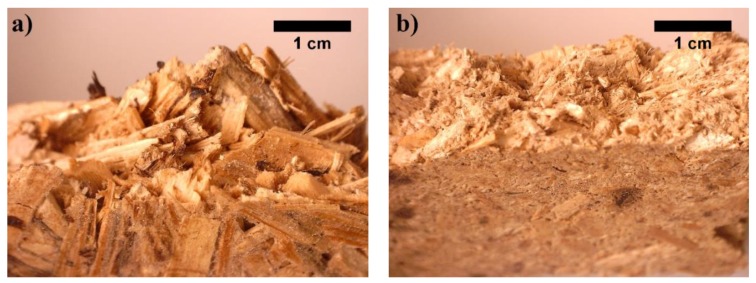
Nature of BcB fracture after bending test (macrostructure): (**a**) from non-shredded HS aggregate; (**b**) from shredded HS aggregate.

The conducted structural analysis shows that fracture surface of BcB from non-shredded HS is uneven (Figure 9a). During bending strength, part of the shivs in a fracture zone partially or totally break. It is as well can be seen that part of the shivs are pulled out. Therefore, it can be stated that connections in these places are not strong enough. Moreover, Figure 9b shows the fracture surface of BcB from shredded HS. The fracture mostly occurs through contact zones of HS while forming quite even fracture line, however, in some places, sole larger particles are pulled out.

It can be noticed that the strength of contact zones between HS aggregate and CS binder is quite uniform throughout the whole specimen volume. Due to the higher amount of contact zoned, which are formed between shredded HS particles, these BcB may withstand higher stresses during the test.

### 3.3. Tensile Strength of BcB

The tensile strength of a material is one of the most important mechanical properties. This parameter is relevant if any other material or product is assigned to be connected or glued on BcB. It is also important during installation or transportation. Based on the value obtained, the application area of the material may be anticipated.

**Figure 10:**
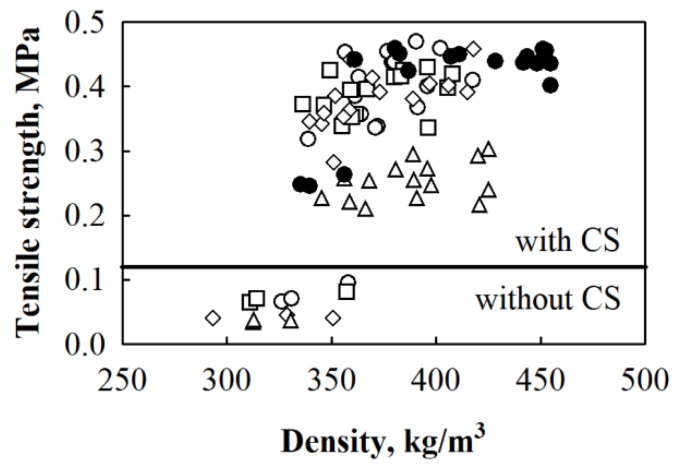
Relationship between tensile strength and density where designated dots present experimental data for BcB from HS fraction, mm: ○—5/10; □—10/20; ◊—2.5/20; Δ—2.5/5; ●—shredded HS; (───)—separation line for results obtained from specimens with and without CS binder.

The conducted experimental data analysis (Figure 10) showed that, according to the tensile strength results, control specimens without a binder may be distinguished into two groups. Control BcB from 5/10, 10/20, 2.5/20 and 2.5/5 mm HS fractions are characterised by the lowest tensile strength with an average value of ~0.057 MPa. Meanwhile, control BcB from shredded HS have the highest value average equal to ~0.25 MPa. With the addition of 10 wt.% of CS binder into the forming mixture, the tensile strength of all HS fraction-based BcB reaches 0.20–0.39 MPa. Due to the porous microstructure of HS, part of the CS particles (<25 μm [37]) can penetrate and fill the vessels and form a good mechanical connection with another part of CS, which then enhance the interface.

**Figure 11:**
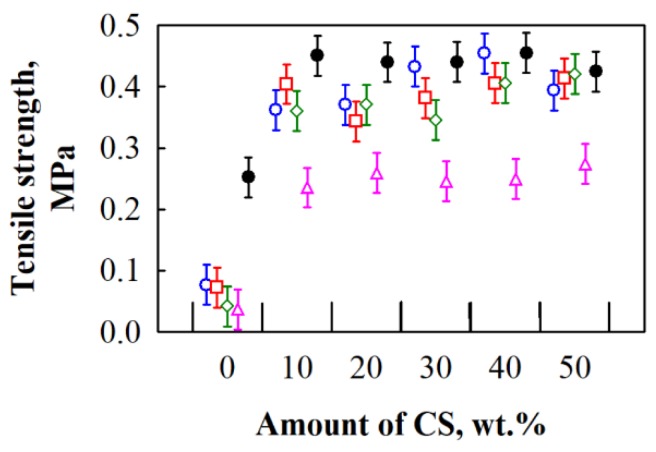
Impact of various amounts of CS and different HS fractions on tensile strength of BcB: ○—5/10 mm; □—10/20 mm; ◊—2.5/20 mm; ∆—2.5/5 mm; ●—shredded HS.

Comparing the results of BcB from all fractions HS and 10 wt.% of CS, it is determined that the tensile strength for shredded HS-based BcB is by ~2.9 times higher than for 2.5/5 mm-based BcB (Figure 11). The similar observation is done by [36] for composites with a smaller fraction aggregate. However, the density range of such composites was from 1100 to 1300 kg/m^3^. Further addition of CS does not significantly change the parameter of BcB from all HS fractions. However, it may be stated that BcB from shredded HS and different CS are characterised by relatively higher tensile strength. As can be seen, the lowest tensile strength is obtained for control BcB without CS binder and the obtained results are in a great agreement with the ones obtained by [38].

**Figure 12:**
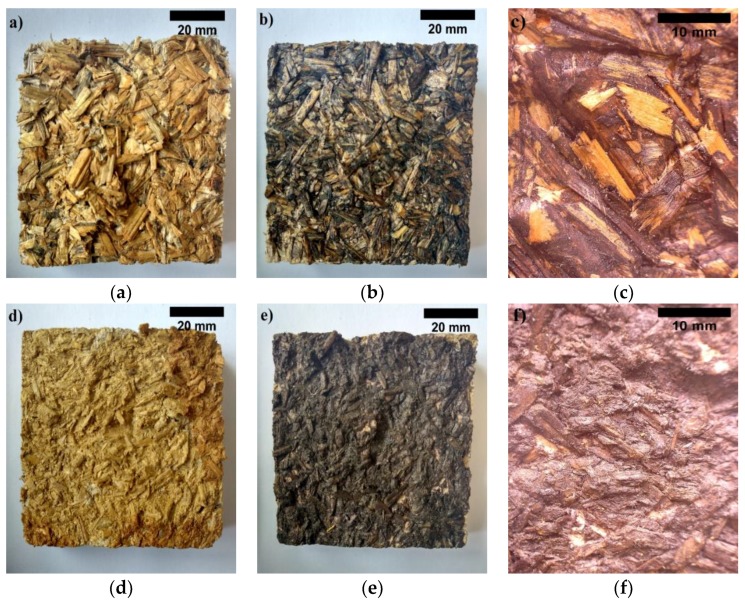
View after tensile test when the BcB are obtained from (magnification ×10): (**a**) non-shredded HS without CS; (**b**) non-shredded HS and 10 wt.% of CS; (**c**) non-shredded HS and 10 wt.% of CS (magnification ×10) (**d**) shredded HS without CS; (**e**) shredded HS with 10 wt.% of CS; (**f**) shredded HS and 10 wt.% of CS.

When the specimen is under tensile force, stresses are formed and they must be compensated by internal cohesive forces between particles. In the case of excess of such forces, the specimen fractures. Therefore, Figure 12 presents fracture zones of BcB after tensile test. Whereas the CS structure consists of strands, which have glucose remaining, iodine molecules may penetrate into them [39]. Consequently, CS when reacted with iodine may colour itself in dark blue colour. In order to evaluate the distribution of CS, a 5% concentration iodine solution was used. Accordingly, Figure 12a,d present the fracture zone of iodine solution coated BcB from 2.5/5 mm and shredded fractions HS without CS. It is seen that the colour of the fracture surface does not change. Contrary observations are completed for iodine solution coated BcB from 2.5/5 mm and shredded fractions HS and 10 wt.% of CS. Therefore, noticeable bright change in colour can be seen (Figure 12b,c,e,f). Comparing Figure 12b,c to Figure 12e,f, it can be noticed that CS binder covers larger area of BcB specimen from shredded HS aggregate. Meanwhile, Figure 12b,c depict light zones, which show that CS binder in BcB from non-shredded HS covers smaller area of specimen compared to the one for BcB from shredded HS.

Contact zones that are not covered with CS may withstand lower stresses while larger specimen surface area covered with CS strengthens contact zones between HS particles. Due to larger cohesive force between particles, specimen is able to sustain higher tensile stresses. This assumption is confirmed by tensile test results. Based on the results obtained, the lowest tensile strength is reached by BcB from 2.5/5 mm HS fraction and it is 0.235 MPa at density of 368 kg/m^3^, while the highest one has BcB from shredded HS and it is 0.451 MPa at density of 375 kg/m^3^. Additionally, in order to better understand the extent of an effect, Table 2 presents mechanical tests results for BcB from HS aggregate and CS binder. The differences emerge due to different production method, raw material preparation, thermal treatment and different densities of materials.

All things considered, the boards with shredded HS fraction and 10 wt.% of CS exhibit good mechanical properties, which make them promising potential substitutes for commercially available products with similar density. The good mechanical performance of BcB can be attributed to the good adhesion between CS and HS. Indeed, as can be observed from the optical images (Figure 12f), the surface of the HS appears to be covered with the binder without significant detachments.

## 4. Conclusions

Based on the results obtained, relationships between the amount of CS binder and mechanical performance were observed. Increasing CS binder amount up to 50 wt.%, increases compressive stress at 10% deformation irrespective of HS fraction used, while improvement in bending and tensile strength can be observed when up to 10 wt.% of CS binder is used. Application of 20–50 wt.% CS does not impact the later properties of BcB.

Overall, regarding the mechanical properties, it is expedient to produce BcB with a shredded HS fraction and 10 wt.% of CS binder under thermal treatment at 160 °C. The thermal process assures the release of lignin from HS. Consequently, the system of lignin and CS strengthens the zones between aggregate particles and form structure, which determines the better mechanical performance compared to BcB with non-shredded HS.

BcB with shredded HS aggregate and 10 wt.% of CS are characterized by the greatest mechanical performance, i.e., independently on HS fraction, such BcB have: compressive stress at 10% of deformation up to 3.0 MPa, bending strength of 6.3 MPa and tensile strength of 0.45 MPa. The obtained average density (~319–408 kg/m^3^) indicates that, according to European normative document EN 316 [40], BcB can be classified as softboards and used as self-bearing structural material for the building industry. Based on the requirements, BcB can be applied in dry and humid conditions for the internal and external uses without loading (EN 622-4, Section 4.2) or as load-bearing boards in dry and humid conditions for instantaneous or short-term load duration (EN 622-4, Section 4.3). 

## Figures and Tables

**Table 1:** The compositions of biocomposite boards.

Raw Materials		HS Fraction, mm
HS, g	CS Binder ^1^, wt.%	
300	0; 10; 20; 30; 40; 50	5/10; 10/20; 2.5/20; 2.5/5; Shredded HS

^1^ the amount of corn starch binder is calculated based on the amount of an aggregate.

**Table 2:** Mechanical properties obtained by different authors and the current research.

Reference	Density, kg/m^3^	Compressive Stress, MPa	Bending Strength, MPa	Tensile Strength, MPa
[21]	182–188	0.57–0.63	–	0.080–0.11
[22]	177–210	0.40–0.80	0.15–0.25	–
[35]	164–174	0.014–0.080	0.030–0.13	–
[36]	300–600	1.2–3.0	0.90–6.8	0.18–0.49
Current results	319–408	1.8–3.4	1.2–6.9	0.092–0.42
